# FUS-mediated functional neuromodulation for neurophysiologic assessment in a large animal model

**DOI:** 10.1186/2050-5736-3-S1-O23

**Published:** 2015-06-30

**Authors:** Wonhye Lee, Hyungmin Kim, Stephanie D Lee, Michael Y Park, Seung-Schik Yoo

**Affiliations:** 1 Brigham & Women’s Hospital, Boston, MA, United States; 2 Korea Institute of Science and Technology, Seoul, Republic of Korea

## Background/introduction

Focused ultrasound (FUS) is gaining momentum as a new modality of non-invasive neuromodulation of regional brain activity, with both stimulatory and suppressive potentials. The utilization of the method has largely been demonstrated in small animals. Considering the small size of the acoustic focus, having a diameter of only a few millimeters, FUS insonification to a larger animal’s brain is conducive to examining the region-specific neuromodulatory effects on discrete anatomical areas, including the white matter (WM) tracts. The study involving large animals would also establish preliminary safety data prior to its translational research in humans.

## Methods

Sheep (all female, 25–40 kg, n = 8) were chosen for the transcranial application of FUS due to their round cranium and skull thickness (4–5 mm) that was close to that of humans. All procedures were approved by the local Institutional Animal Care and Use Committee, and done under the intravenous Tiletamine anesthesia. To provide the information for positioning of the FUS focus to the individual functional neuroanatomy, sheep’s brain was imaged using a 3T MRI scanner (GE VH, Waukesha, WI) using anatomical (both T1- and T2-weighted) and functional MR protocols (fMRI, T2*-weighted). The somatosensory and visual areas of the sheep brain were mapped while using sensorimotor (i.e. gentle 2 Hz squeeze of the right hind leg muscle) and visual (i.e. 2 Hz strobe lights to both eyes) stimuli. As guided by these neuroimage data, the sonication (250 kHz, single-element FUS transducer with radius-of-curvature of 7 cm) was transcranially delivered to the unilateral sensorimotor cortex, the optic radiation (WM tract) as well as the visual cortex. An acoustic intensity of 1.4–15.5 W/cm2 Isppa, tone-burst-duration of 1 ms, pulse-repetition frequency of 500 Hz (i.e. duty cycle of 50%), sonication duration of 300 ms, were used for the stimulation. A batch of continuous sonication ranging from 50 to 150 ms in duration were also given. Evoked electromyogram responses from the hind legs and electroencephalogram from the Fz and Oz-equivalent sites were measured. The histology of the extracted brain tissue (within one week and 2 months post-sonication) was obtained.

## Results and conclusions

The acoustic transmission measured through the extracted skulls (n = 8) was 41.5% of incident intensity. We detected a motor evoked potential (EP) from the hind leg muscle contralateral to the sonicated hemisphere above 6.9 W/cm2 Isppa (i.e. 3.5 W/cm2 Ispta), which served as a threshold intensity. The stimulation was not accompanied by the actual muscle movement. The amplitude of EP was greater than the one obtained using the lower acoustic intensity. Similarly, FUS-mediated visual EP was also detected at a similar threshold level without the presence of external lights. Continuous sonication also elicited stimulatory responses, suggesting that continuous short bursts of the FUS can also stimulate the brain. On the other hand, no response was detected from the sonication of the optic radiation, which indicates that FUS may not stimulate the WM tracts. The sheep’s health status was normal throughout all the sonication experiments. Histological analysis of the extracted brain showed no apparent biological damages. The transcranial FUS may serve as a novel tool to transiently and reversibly modulate regional brain functions, which will enable electrophysiological assessments on the function of an ablative target prior to FUS-mediated neurosurgery.

